# Marine cyanobacterial biomass is an efficient feedstock for fungal bioprocesses

**DOI:** 10.1186/s13068-024-02469-6

**Published:** 2024-02-13

**Authors:** Jai Kumar Gupta, Kavish K. Jain, Mehak Kaushal, Daniel J. Upton, Manish Joshi, Piyush Pachauri, A. Jamie Wood, Syed Shams Yazdani, Shireesh Srivastava

**Affiliations:** 1 Systems Biology for Biofuel Group, International Centre for Genetic Engineering and Biotechnology (ICGEB), ICGEB Campus, Aruna Asaf Ali Marg, New Delhi, 110067 India; 2 DBT-ICGEB Centre for Advanced Bioenergy Research, New Delhi, 110067 India; 3 Department of Biology, University of York, Wentworth Way, York, YO10 5DD UK; 4 Department of Mathematics, University of York, York, YO10 5DD UK; 5 Microbial Engineering Group, ICGEB, New Delhi, 110067 India; 6 Present Address: Zero Cow Factory, Surat, India; 7 Present Address: The Live Green Co., Bangalore, India; 8 Present Address: Perfect Day India Pvt. Ltd., Bangalore, India; 9 Present Address: Biocon Limited, Bangalore, India

**Keywords:** Cyanobacteria, Fungal cellulase, Citric acid, Acid hydrolysis, Sustainability

## Abstract

**Background:**

Marine cyanobacteria offer many sustainability advantages, such as the ability to fix atmospheric CO_2_, very fast growth and no dependence on freshwater for culture. Cyanobacterial biomass is a rich source of sugars and proteins, two essential nutrients for culturing any heterotroph. However, no previous study has evaluated their application as a feedstock for fungal bioprocesses.

**Results:**

In this work, we cultured the marine cyanobacterium *Synechococcus* sp. PCC 7002 in a 3-L externally illuminated bioreactor with working volume of 2 L with a biomass productivity of ~ 0.8 g L^−1^ day^−1^. Hydrolysis of the biomass with acids released proteins and hydrolyzed glycogen while hydrolysis of the biomass with base released only proteins but did not hydrolyze glycogen. Among the different acids tested, treatment with HNO_3_ led to the highest release of proteins and glucose. Cyanobacterial biomass hydrolysate (CBH) prepared in HNO_3_ was used as a medium to produce cellulase enzyme by the *Penicillium funiculosum* OAO3 strain while CBH prepared in HCl and treated with charcoal was used as a medium for citric acid by *Aspergillus tubingensis*. Approximately 50% higher titers of both products were obtained compared to traditional media.

**Conclusions:**

These results show that the hydrolysate of marine cyanobacteria is an effective source of nutrients/proteins for fungal bioprocesses.

**Supplementary Information:**

The online version contains supplementary material available at 10.1186/s13068-024-02469-6.

## Background

Recent reports suggest that approximately 35 billion tons of CO_2_ are released annually from burning of fossil fuels [1]. The rapid increase in atmospheric concentrations of CO_2_ [2] is causing a rise in global temperatures which is detrimental to the world environment. While the world strives to implement policies to mitigate anthropomorphic CO_2_ release into the environment, efforts are needed to develop technologies that can fix atmospheric CO_2_ into usable forms. Oceans absorb approximately 30% of the CO_2_ released into the atmosphere [3] and release 50–80% of the oxygen produced on Earth [4]. This oxygen is produced by marine cyanobacteria, algae and plants.

Marine cyanobacteria offer several other sustainability advantages compared to land-based plants. They have higher photosynthetic efficiency than plants [5], do not require freshwater or arable lands for cultivation [6, 7] and require only minimal media for their growth [6, 8]. Marine cyanobacteria, especially from the *Synechococcus* genus, have been reported as the fastest growing photoautotrophs with doubling times as low as 2.6 h in lab settings using photobioreactors [9, 10]. They also have good tolerance to high salinity, temperature and light [9, 11]. Approximately 70–80% of the cyanobacterial biomass produced is constituted of glycogen and protein. Some previous studies have utilized cyanobacterial glycogen to produce ethanol by culturing yeast in CBH [12, 13]. These studies have used enzymatic treatment to release the sugars from the stored glycogen, which were then fermented by yeast. Enzymatic hydrolysis of cyanobacterial biomass requires longer treatment times. For example, Möllers et al. [12] treated *Synechococcus* biomass with lysozyme for 4–6 h after freezing the biomass for 1 h, followed by 2 h treatment with glucanases at higher temperatures. The cost of enzyme as well as the temperature ranges needed for the complete treatment (− 20 to + 85 °C) are likely to make the enzymatic process economically challenging. Acid and alkaline pretreatments of biomass can hydrolyze the biomass at a higher capacity with shorter reaction times and with greater cost-effectiveness [14–17]. For example, Mustaqim et al. [18] hydrolyzed Synechococcus leopoliensis biomass in 3 N HCl in 20 min at 80 °C while [19] hydrolyzed the biomass of *Scenedesmus obliquus* in 30 min at 120 °C. Acid treatment is a well-established treatment for hydrolyzing sugar polymers as well as for cyanobacterial biomass hydrolysis while treatment with concentrated NaOH is used for estimating protein content of the biomass [20].

In this work, we tested the utility of CBH as a medium for two different fungal bioprocesses: the production of cellulase and citric acid. Cellulases are needed for the hydrolysis of lignocellulosic (LC) biomass, such as wheat and rice straw or sugarcane bagasse, for the sustainable production of biofuels and commodity chemicals. It has been estimated that cellulases can contribute up to 15–30% of the cost of lignocellulosic ethanol [21–23]. *Penicillium* sp., *Trichoderma* sp. and *Aspergillus* sp. are the three major filamentous fungal species used to produce cellulases for lignocellulosic biomass degradation. Proteins added to the media are the major cost contributors for cellulase production. A number of previous studies have focused on the production of cellulases from cost-effective substrates using different microorganisms [24, 25], but there is still a need to explore the use of alternative feedstocks that are sustainable and can potentially reduce the cost of enzyme production.

Citric acid is the most consumed organic acid worldwide and has applications in the food and beverage, pharmaceutical, cosmetics, and chemical industries [26, 27]. Fermentation employing *Aspergillus* strains is the primary mode of production of citric acid [28]. The ability of *Aspergillus* strains to utilize both C5 and C6 sugars makes them ideal candidates for citric acid production. Here, again, the organic nitrogen source is a major determinant of the media costs.

In the present study, we scaled up the culture of *Synechococcus* sp. PCC 7002 to 2 L in an externally illuminated bioreactor. Enzyme-free hydrolysis of the resultant cyanobacterial biomass was optimized for efficient recovery of sugars and/or proteins. We then utilized the treated biomass for cellulase production using *Penicillium* species and citric acid fermentation using *Aspergillus* species. In both cases, higher product titers than the base media were observed, demonstrating the utility of the approach and the potential widespread applicability of cyanobacterial biomass for fungal processes.

## Methods

### Microorganisms used and culture conditions

The cyanobacterium *Synechococcus* sp. PCC 7002 was obtained from Pasteur Culture Collection, (Paris, France), the fungus *Penicillium funiculosum* OAO3 (*Pf*OAO3) [29] was provided by Dr. S. S. Yazdani and *Aspergillus tubingensis* DJU120 G9M7 was provided by Dr. D.J. Upton, University of York). The *Pf*OAO3 strain is a derivative of *Penicillium funiculosum* NCIM1228 with deletion of the catabolite repressor Mig1 and overexpression of cellobiohydrolase 1 and lytic polysaccharide monooxygenase for high levels of cellulolytic enzyme production [29].

### Culture of *Synechococcus* sp. PCC 7002 in an illuminated bioreactor and measurement of glycogen and protein

The cells were grown in A^+^ medium (pH 8.2, composition in the Supplementary File). The total culture volume was 2 L in a 3 L bioreactor (Applikon Biotechnology, Holland). The seeding optical density OD_730_ was 0.1. The reactor was illuminated continuously from outside using customized LED lights (Design Innova, New Delhi, India) [30]. The light intensity was set at 100 µmol m^−2^ s^−1^ at the beginning of the experiment and was gradually increased by 100 µmol m^−2^ s^−1^ every 8 h until the maximum light intensity (1000 µmol m^−2^ s^−1^) and kept at that intensity for the rest of the culture. The bioreactor was bubbled with compressed air at an initial air flow rate of 1 L min^−1^ that was increased to 2 L min^−1^ after 24 h and to 3 L min^−1^ after 48 h and maintained at that rate for the remainder of the run. The impeller speed was maintained at 400 rpm throughout the culture.

Glycogen levels were measured as per our earlier protocols [11]. For the measurement of proteins, 50 mg of lyophilized biomass was hydrolyzed by heating in 5 mL of 1 N NaOH at 95 °C for 5 min [20]. The protein concentration in the supernatant was measured using a BCA Protein Assay Kit (Pierce, Thermo Scientific, Rockford, USA). Bovine serum albumin was used as the protein standard.

### *Penicillium funiculosum* culture and base medium for cellulase production

The seed culture of *Pf*OAO3 [31] was started by inoculating 10^7^ conidiospores in 30 mL of sterile potato dextrose broth (PDB) in 250 mL Erlenmeyer flasks (Borosil, India). The culture was incubated at 28 °C at 150 rpm in an orbital shaking incubator (Innova 44R, New Brunswick) for 36 h. 5 mL of the seed culture were used to inoculate the enzyme-production flask containing 45 mL of RCM medium [31] which was used as the base medium for cellulase production.

### Seed culture of *A. tubingensis*

Seed culture was prepared by inoculating 50 mL seed culture medium with 500 µl of spore stock (10^8^ spores mL^−1^). The medium for seed culture [32] contained glucose (50 g L^−1^), CaCO_3_ (0.03125 g L^−1^), (NH_4_)_2_SO_4_ (0.52 g L^−1^), MnCl_2_·(H_2_O)_4_ (0.0000108 g L^−1^), K_2_HPO_4_ (0.5 g L^−1^), MgSO_4_⋅7H_2_O (0.275 g L^−1^), citric acid monohydrate (3.3 g L^−1^), FeSO_4_⋅7H_2_O (0.0095 g L^−1^), ZnSO_4_⋅7H_2_O (0.00225 g L^−1^), CuSO_4_⋅5H_2_O (0.0117 g L^−1^), urea (3.6 g L^−1^), and Tween 80 (0.0094%). After 2 days, the seed culture was used to inoculate the citric acid production cultures at 10% inoculum volume. The HR medium used as the control medium for citric acid production contained glucose (80 g L^−1^), xylose (40 g L^−1^), vegetable peptone (5 g L^−1^), KH_2_PO_4_ (0.27 g L^−1^), MgSO_4_⋅7H_2_O (0.71 g L^−1^), FeSO_4_⋅7H_2_O (0.015 g L^−1^, ZnSO_4_⋅7H_2_O (0.016 g L^−1^), CuSO_4_⋅5H_2_O (0.011 g L^−1^), MnSO_4_⋅H_2_O (0.0046 g L^−1^), Na_2_SO_4_ (2.4 g L^−1^) and CaCl_2_ (0.27 g L^−1^). The pH was adjusted to 5.0 with 1 N NaOH.

### Enzyme-free hydrolysis of cyanobacterial biomass using acids and base

A 10% loading (w/v) of the biomass was used for all the hydrolysis tests. The biomass was hydrolyzed by the acids at 100 °C or by NaOH at 95 °C. The hydrolysis parameters are summarized in Table 1.

**Table 1:** The factors and their levels tested for hydrolysis of cyanobacterial biomass

Treatment	Acid	Base
Type	HCl, H_2_SO_4_, HNO_3_, H_3_PO_3_	NaOH
Concentration (N)	0.5, 0.75, 1, 2, 3	0.25, 0.5, 0.75
Temperature (°C)	100	95
Time	30, 60, 90, 120	30, 60, 90, 120

Biomass loading of 10% (w/v) was used for all hydrolyses

### Preparing CBH using HCl or HNO_3_

Lyophilized biomass from several fermenter runs was pooled. A total of 30 g cyanobacterial biomass each was taken in two 1 L screw cap glass bottles. Then, 300 mL of acid (1 N HCl or 1 N HNO_3_) was added to the bottles. The mixtures were heated for 90 min in a boiling water bath. Then, the pH was adjusted to ~ 5.0 with NaOH powder (obtained from pellets)*.*

### Cyanobacterial biomass as a feedstock for cellulase production

One gram of the lyophilized cyanobacterial biomass was added to 250 mL Erlenmeyer flasks (Borosil, India), and 10 mL of 1 N HCl, 1 N HNO_3_, 1 N H_2_SO_4_ or 1 N H_3_PO_4_ acids were added. The mixtures were stirred for 20 min on a magnetic stirrer at 200 rpm at room temperature. Other components were added as summarized in Additional file 1: Table S1. The pH was adjusted to 5.5, and the volume was made up to 30 mL by adding Milli-Q water. The flasks were then autoclaved at 121 °C for 20 min. After cooling, 3 mL of inoculum prepared in RCM medium was added to the flasks and the flasks were incubated at 28 °C for 5 days at 150 rpm.

### Measurement of total cellulase activity by the FPU assay

Filter paper unit (FPU) assay was performed for measuring the cellulase activity in the culture supernatant as described earlier [31, 33].

### Charcoal treatment of the HCl^−^ and HNO_3_-treated cyanobacterial biomass (HCl-Char and HNO_3_-Char)

The CBH prepared in HCl or HNO_3_ contained HMF which was thought to inhibit citric acid production. To remove HMF from the CBHs, they were treated with activated charcoal. 0.5 g of activated charcoal (Himedia, Mumbai, India) was added to 100 mL of acid hydrolysates. The mixtures were stirred using a magnetic stirrer for 2 h at room temperature and filtered through Whatman number 1 filter paper. The resulting filtrate was used as the base medium to culture *A. tubingensis*.

### Cyanobacterial biomass as a feedstock for the production of citric acid

To utilize cyanobacterial biomass as a feedstock for the production of citric acid by *A. tubingensis*, CBHs were prepared using different methods as mentioned below. The compositions of the different culture media used are described in detail in Additional file 1: Table S2.

### Supplementary methods

The materials used, basal cyanobacterial culture, culture of *A. tubingensis*, measurement of packed cell volume (PCV), glucose, xylose, citric acid and HMF are given in Additional file 1.

## Results

### Growth, glycogen and protein measurements of *Synechococcus* sp. PCC 7002 in the bioreactor

**Fig. 1:**
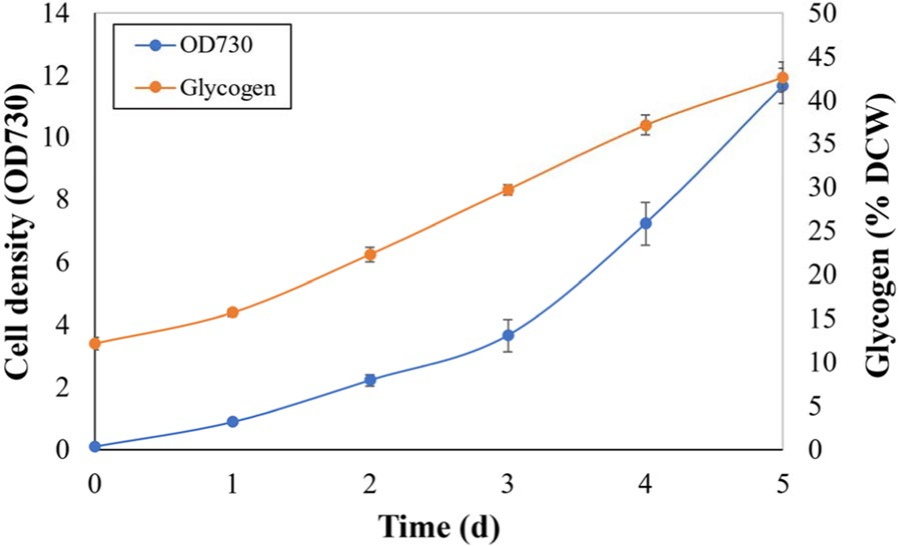
Growth and glycogen content of *Synechococcus* sp. PCC 7002 in an externally illuminated bioreactor. The cells were grown in A^+^ medium with air bubbling. The starting light intensity was 100 μmol m^−2^ s^−1^ and was increased by 100 μmol m^−2^ s^−1^ every 8 h until a light intensity of 1000 μmol m^−2^ s^−1^ was reached. The optical density at 730 nm (OD_730_) of the culture (left *y*-axis) was measured every 12 h, and glycogen content (%, right *y*-axis) was measured every 24 h. *n* = 3 for all measurements

When *Synechococcus* sp. PCC 7002 was grown in the bioreactor with air bubbling, the OD_730_ reached 12 in 5 days (Fig. 1). This corresponds to a biomass productivity of 0.79 g L^−1^ D^−1^. These biomass and glycogen productivities with air bubbling are comparable to those of an earlier study performed in lower volume shake flasks with 1% CO_2_ [13].

It was observed that glycogen content also increased with time. While the glycogen content at the time of inoculation was approximately 12%, it increased to 42.6 ± 1.7% of the dry cell weight (DCW) after 5 days of growth (Fig. 1). Thus, a net glycogen productivity of 0.34 g L^−1^ D^−1^ was obtained at the 2 L culture level. The protein content of the cells at the end of the experiment was 32.0 ± 1.46% of the DCW obtained (measured only for the end-point), giving a productivity of 0.25 g L^−1^ D^−1^. These values of biomass protein and glycogen contents were used to calculate the respective % recovery of the different hydrolysis methods tested.

### Base hydrolysis of biomass

**Fig. 2:**
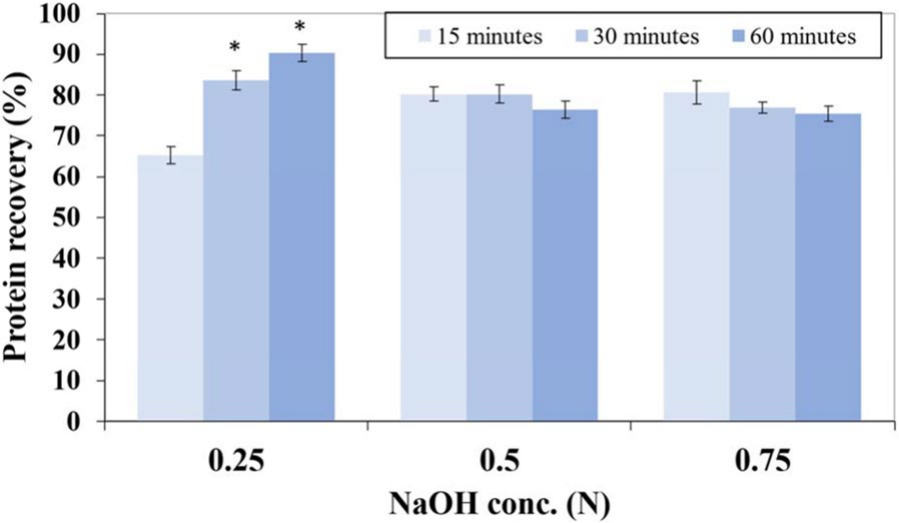
Hydrolysis of the cyanobacterial biomass with NaOH. The recovery of proteins upon hydrolysis of biomass of *Synechococcus* sp. PCC 7002 (10% w/v loading) with different concentrations of NaOH as a function of time. % recovery is based on protein recovered with 1% biomass hydrolyzed in 1 N NaOH for 10 min. * represents a statistically significant difference (*p* < 0.05) in % protein recovery compared to biomass hydrolysis for 15 min for the same NaOH concentration

When the *Synechococcus* sp. PCC 7002 biomass was hydrolyzed by NaOH, proteins, but almost no glucose (not shown), were released. The maximum protein recovery (90.3 ± 2.1%) was observed when the biomass was hydrolyzed with 0.25 N NaOH for 60 min (Fig. 2), while the protein recovery was lower at earlier time-points. A further increase in the concentration of NaOH reduced the time needed to achieve protein recovery but did not significantly increase the protein recovery.

### Acid hydrolysis of cyanobacterial biomass: choice of acid and its concentration

**Fig. 3:**
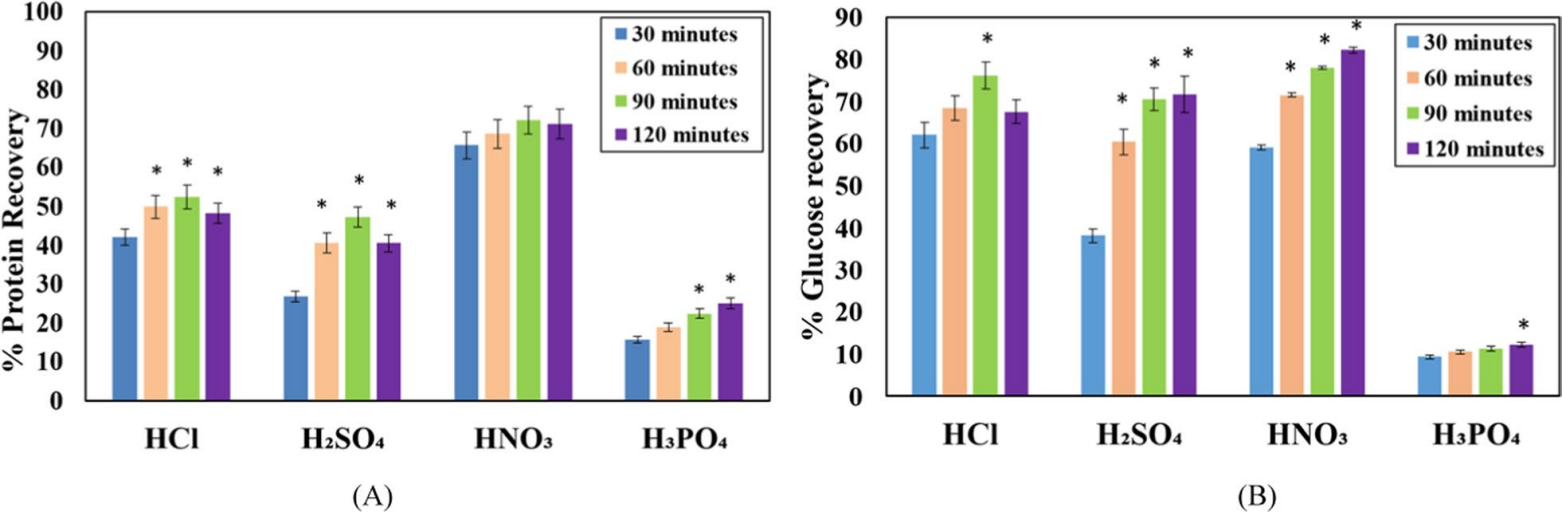
Hydrolysis of the cyanobacterial biomass with acids. Recovery of **A** proteins (in terms of % of total protein) and **B** glucose (in terms of % of total glucose) upon hydrolysis of cyanobacterial biomass with different acids of 1 N strength for different durations. *n* = 3 for all experiments. * represents a statistically significant difference (*p* < 0.05) in biomass hydrolyzed with the same acid for 30 min

Both proteins and sugars were observed in the CBHs upon hydrolysis of the cyanobacterial biomass by acids. Among the various acids tested for hydrolysis, HNO_3_ yielded the highest protein solubilization of up to 78% (Fig. 3A), followed by HCl (protein recovery of 52%). 90 min of treatment was sufficient for the release of proteins in the CBHs (Fig. 3A) with 1 N acids. Increasing the HCl concentration to 2 N reduced the time needed for maximal protein solubilization to 60 min and increased the protein solubilized to 90% (Additional file 1: Fig. S3b). However, increasing the concentration of the other acids did not significantly increase the protein recovery.

Glucose recoveries were comparable among HCl, H_2_SO_4_ and HNO_3_ especially at later time points (Fig. 3B). Here too, HNO_3_ treatment produced slightly higher glucose recoveries compared to other acids. The lowest protein and glucose recoveries were obtained with H_3_PO_4_ (Fig. 3A and B).

Because good recovery of both proteins and sugars was obtained with HNO_3_ hydrolysis of cyanobacterial biomass, further studies with fungal bioproducts were conducted using the CBH prepared in HNO_3_.

### Evaluating cellulase production in cyanobacterial biomass acid hydrolysates

We compared the CBH prepared in various acids for cellulase production by *Pf*OAO3. The highest packed cell volume (PCV) of 27.5% was observed in CBH prepared in H_3_PO_4_ (Table 2), while the lowest PCV (19.3 ± 0.9%) was found in CBH prepared in HNO_3_ (Table 2). In contrast, the highest concentration of extracellular protein was found in the culture supernatant where the fungus was cultured in CBH prepared in HNO_3,_ while culture in the CBH prepared in H_3_PO_4_ resulted in the lowest protein concentration (Table 2).

**Table 2:** The PCV and protein concentration (in mg/mL) obtained in *P. funiculosum* OAO3 cultured in cyanobacterial biomass hydrolysate (CBH) prepared in different acids, and RCM medium (Control)

Culture medium	Packed cell volume (%)	Extracellular protein (mg/mL)
RCM (control)	22.7 ± 0.6	12.6 ± 0.8
HCl-treated CBH	25.1 ± 0.2*	9.9 ± 0.8*
CBH in HNO_3_	19.3 ± 0.9*	14.4 ± 1.2
CBH in H_2_SO_4_	23.9 ± 0.4	9.7 ± 0.3*
CBH in H_3_PO_4_	26.9 ± 0.7*	9.4 ± 0.8*

* indicates a statistically significant difference (*p* < 0.05) in PCV and extracellular protein values of different *P. funiculosum* cultures compared to the RCM (Control) culture

### Cellulase activity in CBH media

**Fig. 4:**
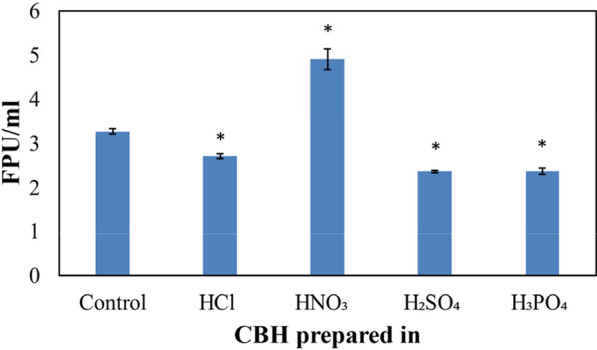
Cellulase production by *P. funiculosum* PfOAO upon culture in CBH prepared in different acids. *P. funiculosum* PfOAO was cultured in the control medium (see “Methods”) or in CBH prepared in different acids. Cellulase activity was measured in the culture supernatants at the end of 5 days of culture using a filter paper unit (FPU) assay. Two biological replicates and three technical replicates for each biological replicate were performed. * represents a statistically significant difference (*p* < 0.05) in FPU mL^−1^ with respect to the control

Culture of the fungus in traditional RCM medium [31] yielded an FPU mL^−1^ of 3.3 ± 0.1. Culture of the fungus in CBH media also led to measurable cellulase activity with all acids. The lowest cellulase activities of 2.4 ± 0.02 FPU mL^−1^ and 2.4 ± 0.07 FPU mL^−1^ were observed in CBHs prepared using H_2_SO_4_ and H_3_PO_4_, respectively. CBH prepared in HCl yielded intermediate levels of FPU mL^−1^ of 2.7 ± 0.06 (Fig. 4). However, in agreement with the increased protein content, the culture of fungus in the CBH prepared in HNO_3_ produced the highest activity of cellulase (4.9 ± 0.2 FPU mL^−1^, Fig. 4). Therefore, the FPU mL^−1^ of the fungal cultures performed in CBHs prepared in HNO_3_ was approximately 50.5 ± 4.3% higher than that of the traditional RCM medium.

### Use of cyanobacterial hydrolysate as the base medium for fermentation of glucose and xylose by *A. tubingensis*

**Fig. 5:**
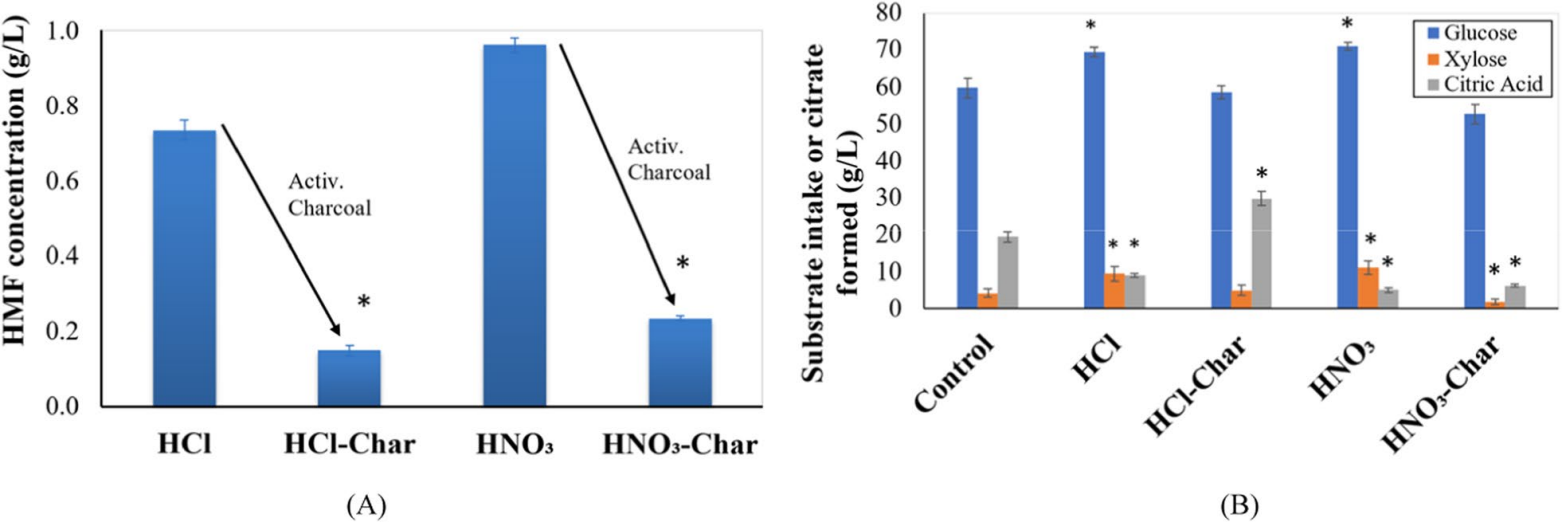
Hydroxymethyl furfural concentration and production of citric acid by the *A. tubingensis* cultures in CBH. **A** Hydroxymethyl furfural (HMF) concentration in CBH prepared and 1 N HCl or HNO_3_ and reduction through treatment with activated charcoal. **B** Consumption of glucose and xylose and release of citric acid by *A. tubingensis* grown in different media for 5 days. Control refers to the HR medium. HCl and HNO_3_ represent cultures in CBH prepared in HCl and HNO_3_, respectively while HCl-Char and HNO_3_-Char represent the cultures in CBH prepared in HCl or HNO_3,_ which was further treated with activated charcoal. Three replicates were performed (*n* = 3). * represents a statistically significant difference (*p* < 0.05) in the consumption of glucose or xylose or the production of citric acid in *Aspergillus tubingensis* cultures performed in cyanobacterial biomass hydrolysate-based media with respect to that in control medium

Acid treatment of sugars, especially at higher temperatures and longer durations, is associated with the production of sugar dehydration products such as hydroxymethylfurfural (HMF). We hypothesized that the presence of inhibitors such as HMF in these media may impact citric acid production. Indeed, CBH prepared by HCl and HNO_3_ treatment contained 73.4 mg ± 3.0 mg L^−1^ and 96.0 ± 2 mg L^−1^ HMF in the CBH, respectively (Fig. 5A). Treatment of the CBH prepared in HCl- or HNO_3_ with activated charcoal reduced the amount of HMF significantly to 15.0 ± 1.0 and 23.0 ± 0.7 mg L^−1^, respectively (Fig. 5A).

Culturing *A. tubingensis* in HR medium for 5 days led to the consumption of 60 g L^−1^ glucose and 4 g L^−1^ xylose and produced 19.3 ± 1.4 g L^−1^ citric acid (Fig. 5B). Culture of the fungus in CBH prepared in HCl led to a higher consumption of both sugars (69 g L^−1^ glucose and 9 g L^−1^ xylose) but produced only 9 g L^−1^ citric acid. Similarly, culture of the fungus in CBH prepared in HNO_3_ also led to a higher consumption of both sugars (71 g L^−1^ glucose and 11 g L^−1^ xylose) but lower citric acid titers (5 g L^−1^). Culture of the fungus in CBH treated with charcoal significantly increased the citric acid produced in the HCl-treated biomass (29.7 ± 1.8 g L^−1^) but not in HNO_3_-treated biomass (Fig. 5B). Thus, approximately 50% more citric acid than that obtained in the charcoal-treated CBH was prepared in HCl than in the control medium.

## Discussion

In this study, we used an illuminated bioreactor to scale-up cyanobacterial cultivation. Both the cyanobacterial biomass and its glycogen content were increasing after 5 days of culture, and it is likely that more biomass could have been obtained with longer culture times. However, as this work is a proof-of-concept of using cyanobacterial biomass for fungal biotechnological processes, we stopped the culture after 5 days of growth, as we had obtained sufficient biomass for subsequent investigations. Second, as both fungal bioprocesses were also run for 5 days, a synchronization of fungal and cyanobacterial culture durations can ease the design of large-scale processes. Utilizing the bioreactor, we could obtain similar biomass productivity with air bubbling as seen in shake flasks with bubbling of 1% CO_2_. It is very likely that biomass and glycogen productivity can be increased further by utilizing higher CO_2_ concentrations and/or engineered cyanobacteria [30, 34]. Alternatively, recently identified marine cyanobacterial strains [35] that show higher basal biomass productivity or glycogen content [36] than *Synechococcus* sp. PCC 7002 could be employed to further increase biomass productivity.

A major energy-consuming step in our cultivation is continuous high-intensity LED illumination. LED lights provide much greater control over the intensity and quality of photosynthetically active radiation (PAR). The LED lights used for such cultivation may be operated using batteries that are recharged using solar energy. This setup, although capital intensive, will reduce the operating costs. In addition, while centrifugation was used here to harvest the culture due to lower volumes, flocculation [36] may also be used to reduce the energy-consuming step of harvest.

Optimal conditions of biomass hydrolysis are also important for the efficient utilization of biomass. We have established simpler hydrolysis methods for the efficient release of both glucose and proteins using acids or for the release of proteins and no glucose using a base (Fig. 2). Thus, for processes that require only proteins, base hydrolysis may be employed. A previous study [14] had used H_2_SO_4_ to hydrolyze algal biomass for subsequent fermentation by *Saccharomyces cerevisiae*. Indeed, H_2_SO_4_ is the cheapest of all the acids tested and as our study also suggests, H_2_SO_4_ is sufficient for releasing sugars for subsequent fermentation. However, HNO_3_ and HCl provide higher recovery of cyanobacterial proteins. HNO_3_ provides an additional advantage in that the remaining nitrate may be used as a nitrogen source by fungi.

Enzymes and organic acids are two major classes of fungal products. For fungal bioprocesses, organic nitrogen (protein) has the greatest contribution to the medium cost. Traditionally, the byproducts of the food processing industry, such as corn steep liquor and soy protein/flour, are used as organic nitrogen sources to minimize this cost. Here, we have shown that for fungal cellulase and citric acid production, the performance of CBH was superior to plant-based proteins. Our work also demonstrates that while cyanobacterial biomass can be a good feedstock for fungal processes, the treatment conditions need to be tailored for a particular product.

Thus, while most previous studies have used cyanobacterial or algal biomass as an efficient sugar source, we demonstrate it as an (equally) efficient protein source. However, some questions regarding the mechanisms of improved performance remain. For example, the increased enzyme production in the HNO_3_-treated CBH-based medium may be explained by the higher protein content of the medium and the presence of nitrate which could have provided an additional nitrogen source. As we got higher enzyme activity with CBH in HNO_3_ (compared to the control medium) without any further treatment, we did not test whether charcoal treatment further improves enzyme production in this medium. This can be tested in future studies. However, the reason for higher citric acid production in charcoal-treated CBH in HCl compared to HNO_3_ is not clear. Future work will examine the mechanisms behind this observation.

In this work, we have tested Synechococcal biomass as a feedstock considering the faster growth of this strain. We have used PBRs for growing these cultures to support fast growth to test the hypothesis. Alternatively, cyanobacterial biomass that is grown on large scale in raceway ponds, such as Spirulina, should also be evaluated similarly. While PBRs provide fast growth rates, allowing quick generation of biomass, overall economic feasibility of PBR vs. raceway ponds is still a topic of active research. For low-cost products, raceway ponds are currently more economically feasible.

## Conclusions

Our results show that base treatment of cyanobacterial biomass releases proteins, while acid treatment releases both glucose and proteins. CBH prepared in HNO_3_ is an effective medium for fungal enzyme production, while CBH prepared in HCl and treated with activated charcoal is an effective medium for citric acid production from a mixture of glucose and xylose. Therefore, the exact treatment of cyanobacterial biomass for subsequent use in fungal processes is dependent on the product desired.

### Supplementary Information

**Additional file 1.** Additional Methods (materials, composition of A^+^ medium, basal cyanobacterial and Aspergillus tubingensis culture, measurement of extracellular protein, glucose, citric acid, hydroxymethylfurfural, different cyanobacterial biomass-based media used for cellulase and citric acid production) and release of proteins and glucose by biomass hydrolysis by different acids of various strengths.

## Data Availability

The datasets used and/or analyzed during the current study are available from the corresponding author upon reasonable request.
