# Supplementary material for: Marine cyanobacterial biomass is an efficient feedstock for fungal bioprocesses
Source: Biotechnol Biofuels Bioprod. 2024 Feb 13;17:23. doi: 10.1186/s13068-024-02469-6 (PMC10863111; doi:10.1186/s13068-024-02469-6)
Supplement: Supplementary file 1 — Additional file 1. Additional Methods (materials, composition of A+ medium, basal cyanobacterial and Aspergillus tubingensis culture, measurement of extracellular protein, glucose, citric acid, hydroxymethylfurfural, different cyanobacterial biomass-based media used for cellulase and citric acid production) and release of proteins and glucose by biomass hydrolysis by different acids of various strengths. [file 13068_2024_2469_MOESM1_ESM.docx]

**Additional data file**

**“Cyanobacterial biomass is an efficient feedstock for fungal processes”**

**Additional Methods**

*Materials*

The medium components used in the study were of analytical grade and purchased from Sigma‒Aldrich Chemical Company (NaCl, MgSO_4_·7H2O, Na_2_EDTA.2H_2_O, CaCl_2_·2H_2_O, NaNO_3_, KH_2_PO_4_, NaOH, HCl, HNO_3_, H_2_SO_4_ H_3_PO_4_, CuSO_4_·5H_2_O, H_3_BO_3_, vitamin B_12_, FeCl_3_·6H_2_O, MnCl_2_·4H_2_O, ZnCl_2_, CoCl_2_·6H_2_O), Thermo Fisher Scientific (Tris base, KCl) and SLR Fisher (Glucose). Suppliers of other chemicals, reagents and instruments are mentioned below.

*Basal cyanobacterial culture conditions*

A^+^ medium (pH 8.2) was used for culturing and scale-up. The culture was maintained at 30 °C under 150 μmol m^−2^ s^−1^ with a light: dark cycle of 16:8 h in an incubator shaker (Innova 44R, New Brunswick) at 150 rpm.

*A^+^ medium composition*

The detailed procedure for preparation of A+ medium is available at <https://utex.org/products/a-plus-medium>

It contains the following components

| **S.No.** | **Component** | **Final Concentration (mM)** |
| --- | --- | --- |
| 1 | NaCl | 0.308 M |
| 2 | MgSO_4_**•**7H_2_O | 0.02 M |
| 3 | Na_2_EDTA•2H_2_O | 0.08 mM |
| 4 | KCl | 8.05 mM |
| 5 | CaCl_2_**•**2H_2_O | 2.52 mM |
| 6 | NaNO_3_ | 11.8 mM |
| 7 | KH_2_PO_4_ | 0.37 mM |
| 8 | Tris Base pH 8.2 | 8.26 mM |
| 9 | [A+ Trace Components (sterilize before adding)](https://utex.org/products/a-plus-trace-components) | 10 mL/L |

*A+ Trace Components Recipe*

| **S.no.** | **Component** | **Final Concentration** |
| --- | --- | --- |
| 1 | H_3_BO_3_ | 55.5 mM |
| 2 | ZnCl_2_ | 0.23 mM |
| 3 | MoO_3_**(85%)** | 0.021 mM |
| 4 | Vitamin B_12_ (cyanocobalamin) | 0.3 µM |
| 5 | FeCl_3_**•**6H_2_O | 0.14 mM |
| 6 | MnCl_2_**•**4H_2_O | 0.22 mM |
| 7 | CuSO_4_**•**5H_2_O | 0.00012 mM |
| 8 | CoCl_2_**•**6H_2_O | 0.0005 mM |

*Aspergillus tubingensis and culture conditions*

*Aspergillus tubingensis* DJU120 G9M7 was cultured in 30 mL medium in 250 mL Delong neck baffled bottom shake flasks (Chemglass Life Sciences, New Jersey, United States) at 30 °C and 250 rpm in an orbital shaking incubator (MRC Laboratory Instruments).

*Measurement of extracellular protein*

*Pf*OAO3 cultures in RCM or CBH media were harvested after 5 days by centrifugation at 9000 × g for 10 minutes at room temperature. The protein concentration in the supernatant was measured using a BCA assay kit (Pierce, Thermo Scientific, Rockford, USA).

*Measurement of glucose, xylose and citric acid*

At the end of 5 days, *A. tubingensis* cultures were centrifuged at 9000 × g for 10 minutes at room temperature. The supernatants were diluted and filtered through 0.22 µm filters (Nylon-66, MDI Membrane Technologies, India). Citric acid, glucose, and xylose concentrations in the supernatants were measured by HPLC (Agilent Technologies) using an Aminex HPX-87H column. Sulfuric acid (5 mM) was used as the mobile phase at a flow rate of 0.3 ml min^−1^.

*Measurement of hydroxymethylfurfural (HMF) using HPLC*

The CBHs were analyzed for the major furan inhibitors, viz. furfural and hydroxymethylfurfural (HMF) using HPLC (Agilent Technologies) with an Aminex HPX-87H column and refractive index detector (RID). The column temperature was maintained at 60 °C. H_2_SO_4_ (4 mM) was used as the mobile phase at a flow rate of 0.6 mL min^-1^.

**Table S1.** The table shows the different cyanobacterial biomass-based media used for the culture of *P. funiculosum OAO3* for cellulase production

| **S.No.** | **Components** | **HCl Treated** | **HNO_3_ Treated** | **H_2_SO_4_ Treated** | **H_3_PO_4_ Treated** |
| --- | --- | --- | --- | --- | --- |
| 1 | Cyanobacterial Biomass (g) | 1 | 1 | 1 | 1 |
| 2 | 1N HCl (mL) | 10 | - | - | - |
| 3 | 1N HNO_3_ (mL) | - | 10 | - | - |
| 4 | 1N H_2_SO_4_ (mL) | - | - | 10 | - |
| 5 | 1N H_3_PO_4_ (mL) | - | - | - | 10 |
| 7 | Tween 80 (µL) | 30 | 30 | 30 | 30 |
| 8 | Wheat Bran (mg) | 642 | 642 | 642 | 642 |
| 9 | MCC (mg) | 720 | 720 | 720 | 720 |

Note: The volume was made up to 30 ml, pH was adjusted to 5.5 and the media were then autoclaved at 121 °C for 20 min.

**Table S2.** The requirements to prepare 30 mL media used to culture *A. tubingensis* for citric acid production.

| **Media Components** | **HCl** | **HCl-Charcoal** | **HNO_3_** | **HNO_3_-Charcoal** |
| --- | --- | --- | --- | --- |
| CBH prepared in HCl | 10 mL | - | - | - |
| CBH prepared in HCl treated with activated charcoal | - | 10 mL | - | - |
| CBH prepared in HNO_3_ | - | - | 10 mL | - |
| CBH prepared in HNO_3_ treated with activated charcoal | - | - | - | 10 mL |
| Adjust pH to 5.0. Make up the volume to 18 ml. Autoclave the media (in delong neck baffled bottom 250 ml shake flasks) at 121 ºC for 20 minutes. | | | | |
| 5X Glucose stock (400 g∙L^-1^) | 6 mL | 6 mL | 6 mL | 6 mL |
| 5X Xylose stock (200 g∙L^-1^) | 6 mL | 6 mL | 6 mL | 6 mL |

**Measurement of protein recovery in acid hydrolyzed cyanobacterial biomass**

**Figure S3a**

**Figure S3b**

*

*

**Figure S3c**

**Figure S3.** Release of proteins (in terms of percent protein recovery) by biomass hydrolysis by different acids of various strength **(S3a)** 0.5 N, **(S3b)** 2 N and **(S3c)** 3 N strength as a function of time.

**Measurement of glucose recovery in acid hydrolysed cyanobacterial biomass**

**Figure S4a**

**Figure S4b**

**Figure S4c**

**Figure S4.** Release of glucose (in terms of percent glucose recovery) by biomass hydrolysis by different acids of various strength **(S4a)** 0.5 N, **(S4b)** 2 N and **(S4c)** 3 N strength as a function of time.
